# Production of Silver Nano-Inks and Surface Coatings for Anti-Microbial Food Packaging and Its Ecological Impact

**DOI:** 10.3390/ijms24065341

**Published:** 2023-03-10

**Authors:** N. Arul Manikandan, Ronan McCann, Dimitrios Kakavas, Keith D. Rochfort, Sithara P. Sreenilayam, Godze Alkan, Tom Stornetta, Allan Robert McGivern, Konstantinos Grintzalis, Bernd Friedrich, Greg Foley, Dermot Brabazon, Brian Freeland

**Affiliations:** 1 School of Biotechnology, Dublin City University, Glasnevin, D09 K20V Dublin, Ireland; 2 I-Form, Advanced Manufacturing Research Centre, School of Mechanical and Manufacturing Engineering, Dublin City University, Glasnevin, D09 NA55 Dublin, Ireland; 3 Advanced Processing Technology Research Centre, School of Mechanical and Manufacturing Engineering, Dublin City University, Glasnevin, D09 NA55 Dublin, Ireland; 4 Department of Science, School of Science and Computing, South East Technological University, Cork Road, X91 K0EK Waterford City, Ireland; 5 School of Nursing, Psychotherapy and Community Health, Dublin City University, Glasnevin, D09 NA55 Dublin, Ireland; 6 Institute of Process Metallurgy and Metal Recycling (IME), RWTH Aachen University, Intzestraße 3, 52056 Aachen, Germany

**Keywords:** antibacterial, food packaging, laser ablation, silver nanoparticles, ultrasound pyrolysis

## Abstract

Food spoilage is an ongoing global issue that contributes to rising carbon dioxide emissions and increased demand for food processing. This work developed anti-bacterial coatings utilising inkjet printing of silver nano-inks onto food-grade polymer packaging, with the potential to enhance food safety and reduce food spoilage. Silver nano-inks were synthesised via laser ablation synthesis in solution (LaSiS) and ultrasound pyrolysis (USP). The silver nanoparticles (AgNPs) produced using LaSiS and USP were characterised using transmission electron microscopy (TEM), Fourier transform infrared (FTIR) spectroscopy, UV-Vis spectrophotometry and dynamic light scattering (DLS) analysis. The laser ablation technique, operated under recirculation mode, produced nanoparticles with a small size distribution with an average diameter ranging from 7–30 nm. Silver nano-ink was synthesised by blending isopropanol with nanoparticles dispersed in deionised water. The silver nano-inks were printed on plasma-cleaned cyclo-olefin polymer. Irrespective of the production methods, all silver nanoparticles exhibited strong antibacterial activity against *E. coli* with a zone of inhibition exceeding 6 mm. Furthermore, silver nano-inks printed cyclo-olefin polymer reduced the bacterial cell population from 1235 (±45) × 10^6^ cell/mL to 960 (±110) × 10^6^ cell/mL. The bactericidal performance of silver-coated polymer was comparable to that of the penicillin-coated polymer, wherein a reduction in bacterial population from 1235 (±45) × 10^6^ cell/mL to 830 (±70) × 10^6^ cell/mL was observed. Finally, the ecotoxicity of the silver nano-ink printed cyclo-olefin polymer was tested with daphniids, a species of water flea, to simulate the release of coated packaging into a freshwater environment.

## 1. Introduction

The growth of microbes in food and resulting food spoilage due to microbial growth is a well-known concern worldwide, particularly in terms of sustainability and food security. Annually, food-borne diseases contribute to 600 million illnesses worldwide, resulting in 420,000 deaths [1]. This highlights the need for advanced packaging solutions with anti-microbial properties that can inhibit microbial growth and extend shelf-life, thus reducing food spoilage [1,2]. Around the globe, antimicrobial packaging films were developed by adding organic or inorganic materials with antimicrobial properties into polymeric matrices [3,4]. The stable performance of inorganic materials amidst varying temperatures and harsh working conditions has made inorganic materials (nanoparticles) outperform conventional organic antimicrobial compounds such as essential oils or plant extracts [4,5]. Furthermore, using nanomaterials is intriguing as it does not alter the organoleptic properties, aroma and flavour of the food products [5,6]. The use of silver nanoparticles (AgNPs) as an inorganic antimicrobial agent is widely accepted due to their broad spectrum of antibacterial activity, heat resistivity, low cytotoxicity and the resulting extended shelf life of packaged foods [5,6]. Though the AgNPs exhibit antimicrobial activity through the continuous release of Ag^0^ and Ag^+^ species, it has been reported in the literature that the size and morphology of the nanoparticles could significantly influence the antibacterial activity of the AgNPs [5,6].

Thus, producing AgNPs of uniform size with a smaller diameter and larger surface area exhibiting superior antibacterial activity is essential. As a result, the preparation of silver nanoparticles having uniform size without any change in the surface properties is considered a key area of research in nanotechnology. Top-down approaches for nanoparticle synthesis, such as laser ablation synthesis in solution (LASiS) and ultrasonic spray pyrolysis (USP), are reported to be simpler and more scalable than the bottom-up wet preparation technique, which involves the use of hazardous chemicals [7,8]. The laser-assisted synthesis (LASiS) technique utilises a high-power laser to ablate metal from a target plate [7]. The ablated material then condenses to form nanoparticles, resulting in a well-defined colloid [9,10]. In contrast to the LASiS technique, ultrasonic spray pyrolysis (USP) utilises ultrasound to atomise liquid droplets, which are then evaporated and thermally reduced to form the desired nanoparticles [8,11]. Both of the aforementioned techniques were tested in the present study, and their performance was compared in terms of having reasonable control of particle size, morphology and surface chemistry of the nanoparticles.

The inherent characteristics of polymers, such as mouldability, durability, ease of manufacturing and high mechanical efficiency, have made polymeric material an inevitable alternative in the packaging sector [12]. Therefore, incorporating silver nanoparticles in the polymer to prepare antimicrobial packaging films is prevalent in the literature [5,6]. However, the negative effects of silver nanoparticles leaching onto food items is a matter of great concern and a topic of ongoing research [5]. In that regard, the present study proposes the preparation of silver nanoparticles to protect food from pathogens while inkjet printing them on the outer surface of the polymer to restrict the intrusion of silver nanoparticles into food products. Nevertheless, food spoilage due to the inherent presence of microbiota in food is inevitable. Advanced food preservation techniques to inhibit such food-borne microbes is an exciting area of ongoing research, but beyond the scope of this article.

Ease of operation, suitability to work on different polymeric matrices, and environment friendliness with reduced waste generation propose inkjet printing as a suitable technique for coating nanoparticles having antimicrobial properties [13]. To date, inkjet printing on food packages has been limited mainly to the inline printing of product codes, manufacturer details and expiry dates. Therefore, preparing silver nano-ink and coating it on polymeric surfaces through inkjet printing will ensure ease of operation and commercial scalability of the antibacterial packaging process [13]. As for polymer matrix concerns, various polymers are used for packaging food items. However, the increasing safety awareness and expanding area of polymer research introduce new polymers such as cyclo-olefin to develop medical devices and food containers [14,15]. The use of cyclo-olefins for producing antimicrobial packaging film is particularly intriguing because of high transparency, high chemical resistance, low birefringence, low specific gravity and low water absorbance [14,15]. Unlike other polymers, cyclo-olefin does not emit endocrine-disrupting chemicals. Therefore, it is found to be safe for human health. Further, cyclo-olefin is also well-known for its use in producing feeding bottles for babies and tableware for school children [15].

The novelty of the present study is the use of cutting-edge laser and ultrasonic production systems for colloid synthesis and printing it on cyclo-olefin polymer for the production of antibacterial packaging films. To the best of our knowledge, such a novel approach was not reported in the literature. Furthermore, the present work aims to compare the properties of AgNPs produced by LASiS and USP via transmission electron microscopy (TEM), UV-visible spectroscopy, dynamic light scattering (DLS) and Fourier transform infrared (FTIR) spectroscopy analysis and their antibacterial effect on *E. coli* cells. The viscosity of silver nano-ink was optimised for its use in inkjet printing by varying the ratio of isopropyl alcohol and deionised water containing AgNPs. Finally, the silver nano-ink was printed on cyclo-olefin polymer and subjected to antibacterial using *E. coli* and ecotoxicity analysis using daphniids (*Daphnia magna*). Thus, the present study strives to increase the knowledge of the sustainable production of AgNPs followed by its coating using inkjet printing and its ecotoxic effect on the water ecosystem.

## 2. Results and Discussion

### 2.1. Characterisation of Silver Nanoparticles

**Figure 1:**
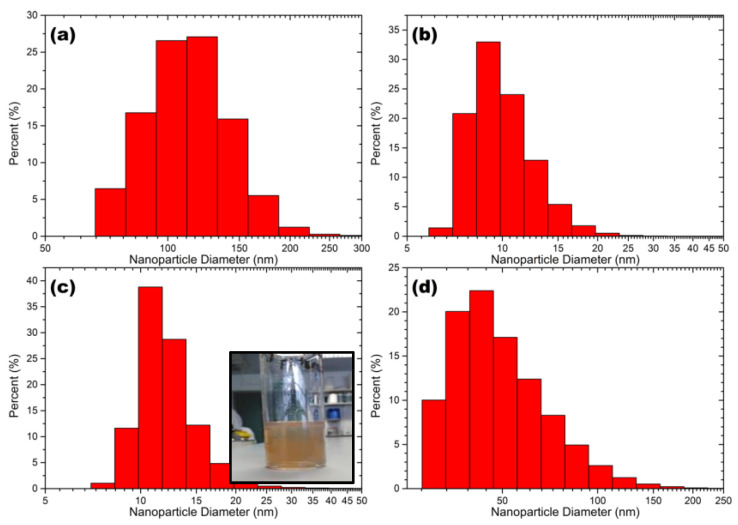
DLS nanoparticle size distributions for AgNPs produced via (**a**) USP, (**b**) commercial AgNPs (Sigma Aldrich), (**c**) recirculatory LASiS and (**d**) batch LASiS produced.

Figure 1 shows the size distributions of the AgNPs produced via USP (Figure 1a), LASiS operated under recirculation mode (Figure 1c) and LASiS operated under batch mode (Figure 1d). Amongst all the three nanoparticle synthesising strategies, NPs produced via LASiS under recirculation mode showed a narrow particle size distribution with the smallest mean size. For instance, AgNPs produced via ultrasound technique displayed a particle size of 115 (±45) nm and AgNPs produced by LASiS technique under batch mode (Figure 1d) had a size of 45 (±40) nm. In contrast, those produced via LASiS operated under recirculation mode (Figure 1c) had a nanoparticle with an extremely smaller size of 12 (±10) nm. The smaller nanoparticle size seen for recirculatory flow-based ablation compared to batch can be attributed to zero-dimensional ablation of nanoparticles as the colloid is recirculated over the ablation material. This process reduces the size of the nanoparticles as a function of ablation time. The size range of silver nano-ink produced by USP is comparable to NPs produced via wet-chemical and biological techniques [16].

**Figure 2:**
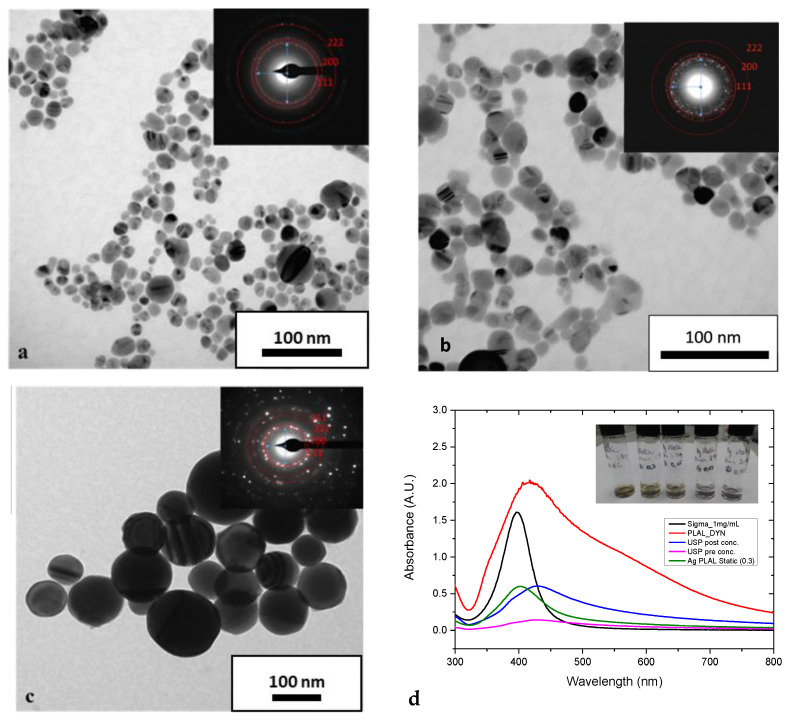
TEM shows typical TEM bright field micrographs and the corresponding selected area diffraction patterns of (**a**) batch LASiS prepared AgNPs, (**b**) recirculatory flow LASiS produced AgNPs and (**c**) USP prepared Ag nanoparticles. (**d**) UV–VIS absorption spectra of AgNPs colloid produced by LASiS Batch recirculatory flow, USP and commercial wet-chemical synthesis, USP post-concentration with rotavapor. LASiS experiments with fPRF = 10 kHz and v = 5 mm s^−1^. The inset shows the AgNPs colloids generated by batch LASiS.

Figure 2 shows TEM images and bright field micrographs of the various colloids produced. Nanoparticles from both production techniques exhibited a spherical morphology. However, the NPs produced via USP were seen to have less ellipticity than those produced via LASiS. It is evident from the bright field micrographs of AgNPs produced by both batch and recirculatory flow mode. The powder diffraction pattern (insets in the bright field micrographs in Figure 2) obtained from the particles prove their phase purity as FCC Ag. The particles produced by the USP process are dense and nearly ideally spherical. Diffraction analysis confirms that the particles produced by USP are crystalline FCC Ag. A higher number of powder diffraction rings visible in the USP-prepared sample than in the LASiS samples are the larger particles, which translates into a higher diffraction volume that increases the diffraction intensity. Nanoparticles produced by both processes, LASiS and USP, exhibit lattice defects as evident from deviations in the Bragg diffraction, i.e., diffraction contrast, inside individual particles. Such lattice defects might have formed due to the high heating and cooling rates during pyrolysis. It is speculated that the surface facets of particles produced by LASiS result from energy minimisation approaching a thermodynamic equilibrium-driven shape [17]. This might be caused by the short crystal growth time due to the high cooling rates [8,18].

Figure 2d shows the UV-Vis absorption spectra of the obtained colloidal solutions. All absorption spectra show the peaks in the ~400 nm wavelength region. The location of this peak indicates surface plasmon resonance peaks due to metallic AgNPs. Nano colloids of smaller particle size distribution absorb the incident light and exhibit absorption peaks around 400 nm wavelength region. However, the colloids consisting of more significant particle size distribution creates light scattering and form broad absorption peak and shift toward the longer wavelength leading to a red shift phenomenon [19,20]. Such red shift was not explicitly observed for any nanoparticle produced in the present study. The UV-Vis spectra of the AgNPs produced in the present study compare well with that of the commercial AgNPs procured from Sigma–Aldrich^®^ (Arklow, Ireland). From Figure 2d, it can also be observed that no secondary peaks were observed for all the nanoparticles produced USP and by LASiS operated under batch and recirculation mode. The absence of secondary peaks shows the stability of AgNPs in the solution [21].

**Figure 3:**
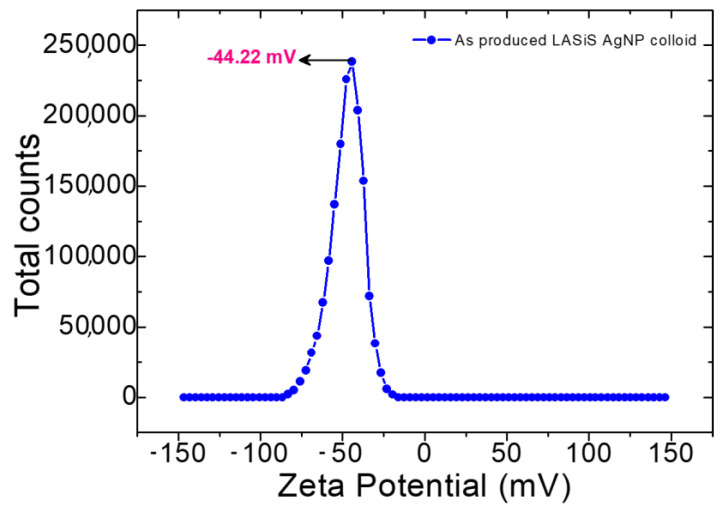
Zeta potential of as produced LASiS AgNPs.

Figure 3 shows the zeta-potential (ξ) measurements of the as-produced LASiS AgNPS colloid in DI water. Nanoparticle stability in suspension depends on this physical property, ξ, which is influenced by surface charges. Therefore, ξ determination is very important for nano colloids. Due to electrostatic repulsion between each individual NPs, colloids with a large positive/negative ξ value generally indicate good stability [9,20]. A small ξ value indicates the Nps aggregation due to the van der Waals attractive forces, leading to colloidal instability [9,19]. The ξ value for LASiS AgNPs colloid produced in this study was −44.22 mV, indicating high colloidal stability.

**Figure 4:**
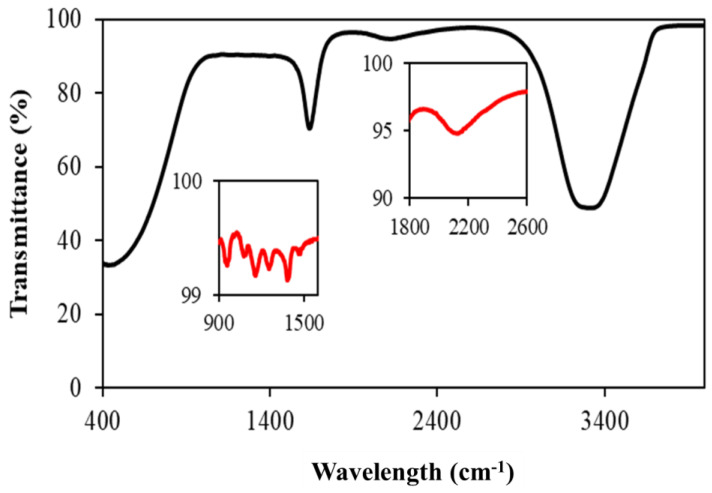
FTIR analysis of silver nano ink used for printing on the cyclic olefin polymer surface. FTIR analysis of the dried AgNPs was provided as the inset.

The functional groups associated with the surface of metallic nanoparticles were characterised using Fourier transform infrared spectroscopy. Figure 4 shows the clean surface of AgNPs with very little oxidation of silver. The bands observed at 3292 cm^−1^ and 2095 cm^−1^ were due to the –OH group and overtones. The bands at 1647, 1395 and 1254 cm^−1^ were due to the carbonate complexes of the Ag-O compounds. The presence of weak carbonate compounds shows that the AgNPs were slightly oxidised. The functional groups in this study closely matched the FTIR spectra of silver nanoparticles ablated by varying the laser parameters [22].

### 2.2. Antibacterial Activity of Silver Nanoparticles

**Figure 5:**
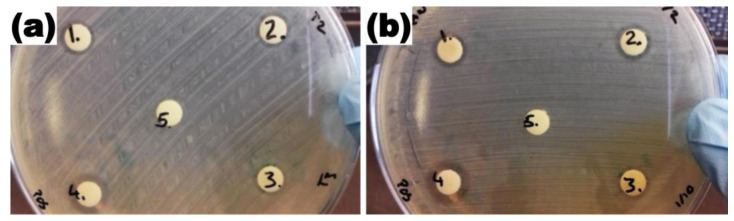
(**a**) Concentrated USP triplicates (**1**), one-in-two dilution (**2**) and one-in-ten dilutions (**3**), commercial AgNPs (**4**) and DI water (**5**). (**b**) Zones of inhibition for LASiS recirculatory flow AgNPs concentrated (**1**), one-in-two dilution (**2**) and one-in-ten dilutions (**3**), commercial AgNPs (**4**) and DI water (**5**).

The antibacterial activity of AgNPs produced through ultrasound pyrolysis techniques and LaSiS technique was tested against *E. coli*. As envisaged, the size of the AgNPs affected their antibacterial activity against *E. coli*. AgNPs produced following ultrasound pyrolysis resulted in a nanoparticle having a larger particle diameter, and therefore, its zone of inhibition at 300 mg/L concentration was relatively low with an inhibition zone of 6.34 mm (Figure 5). Consequently, the smaller diameter of AgNPs produced through LASiS technique operated under recirculation mode with 300 mg/L concentration resulted in a higher zone of inhibition of 6.45 mm. Additionally, as anticipated, no inhibition was observed when this study used deionised water as the negative control. Inclusion of a positive control using commercially available AgNPs with 20 mg/L exhibited a high zone of inhibition of 7.15 mm (Table 1).

**Figure 6:**
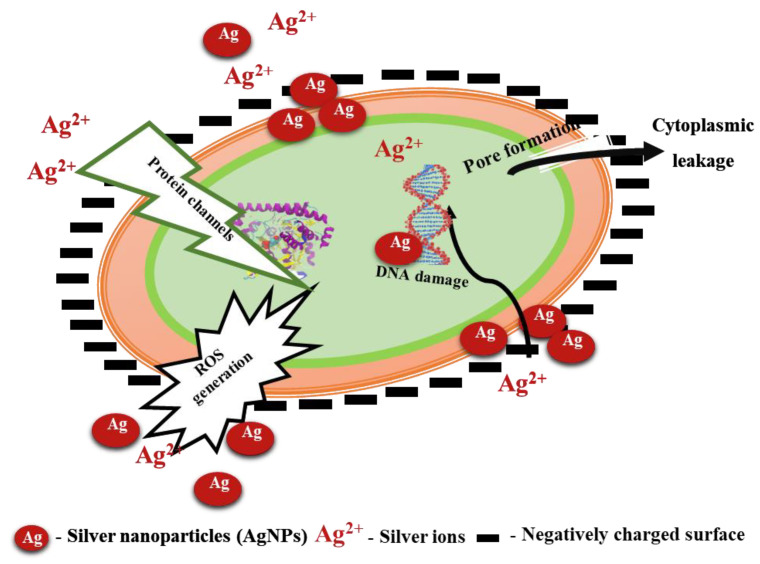
Mechanism involved in the bactericidal activity of silver nanoparticles (AgNPs) due to its nano and ionic attributes. DNA—deoxyribonucleic acid and ROS—reactive oxygen species.

Figure 6 portrays the mechanism of the antibacterial effect of AgNPs on *E. coli*. The enhanced bacterial inactivation with AgNPs can be attributed to the direct penetration of AgNPs into the cytoplasm through protein channels or by forming pores on the cell wall of the microorganisms. The presence of AgNPs in the cytoplasm increases ROS generation causing damage to nucleic acid and protein structure [23]. In addition to the direct penetration of AgNPs, the conversion of AgNPs into silver ions was also reported to cause microbial cell death, as the positively charged silver ions disrupt the negatively charged cell wall leading to leakage of cellular contents [3,5]. Thus, the cumulative effect of reduced AgNPs size produced through the LASiS technique operated under batch recirculation mode and the formation of silver ions could have resulted in a better inhibition of *E. coli.*

### 2.3. Rheological Properties of Silver Nano-Ink

**Figure 7:**
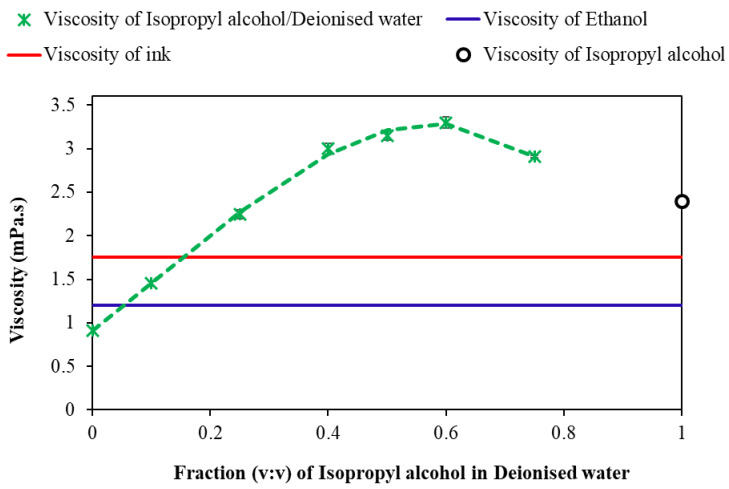
Dynamic viscosity tests of isopropanol/deionised water-fractions, inkjet commercial ink and ethanol. The white band (bounded by the horizontal blue and red lines) on the graph indicates the dynamic viscosity range limit for successful printing.

The property of silver nano-ink play a vital role in inkjet printing, particularly the viscosity of the ink plays a crucial role in the printing process. The viscosity of the ink is fixed between 1 and 20 mPa.s to avoid excessive fluidity or ink blockage in the printer head [10]. The viscosity of the silver nano-ink was adjusted to its desirable value by mixing isopropanol with deionised water in varying ratios. Figure 7 shows the variation in viscosity of silver nano-ink with an increasing ratio of isopropanol in deionised water. In this work, the viscosity of the as-produced AgNPs colloids was tuned to meet commercially used inkjet fluids by mixing AgNPs colloids with different solvents. Increasing the volumetric ratio of isopropanol in deionised water from 0 to 0.6 increased the viscosity of the solution from 0.91 (±0.02) mPa.s to a maximum of 3.3 (±0.062) mPa.s. Further increase in the isopropanol ratio to 0.75 in deionised water reduced the viscosity from 3.3 (±0.062) to 2.91 (±0.01) mPa.s. To replicate the viscosity of the commercial ink at 1.75 mPa.s, this viscosity of the silver nano-ink was achieved by blending 18% of isopropanol with 82% of deionised water containing AgNPs. The non-linear variation of viscosity with an increase in the volumetric ratio of isopropanol agrees with the previous results reported in the literature, wherein the viscosity of carbon nanoparticles was controlled by the addition of isopropanol for printing application [10].

### 2.4. Inkjet Printing of Silver Nano-Ink on Cyclo-Olefin Polymer (COP)

**Figure 8:**
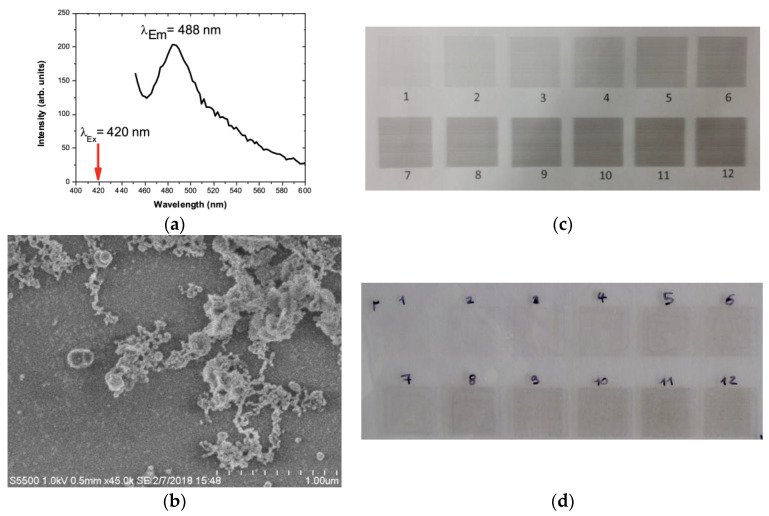
(**a**) Photoluminescence analysis of AgNPs produced recirculatory-flow LASiS showing the appearance of a fluorescence peak at 488 nm (excitation wavelength 420 nm). (**b**) FE-SEM analysis of silver nano-ink-coated cyclo olefin, AgNPs ink (18% isopropanol, 82% AgNPs containing deionised water) overprint trials on polymer (**c**) cellulose and (**d**) plasma cleaned cyclo-olefin polymer sheets (COP). The numbers indicate the number of overprints on the same square print area.

Inkjet printing utilises a concentrated jet of atomised inks deposited on the desired location of the polymeric surfaces to impart desired property of the ink onto the polymeric surfaces [13]. In the present study, silver nano-ink being an inorganic antimicrobial compound, deposition of silver in the polymeric surfaces imparted antibacterial properties to the polymeric surfaces [4]. The photoluminescence spectra of the silver nano particles ablated with pulsed laser operated under recirculatory mode is displayed in Figure 8a. A strong peak at 488 nm (blue emission) was obtained employing an excitation wavelength of 420 nm. Defect emissions due to lattice effects were reported at 488 nm and this corroborates with Bragg diffraction analysis using TEM. The presence of AgNPs on the surface of cyclo olefin polymer was visualised using FESEM (Figure 8b). Plasma treatment was carried out to turn COP hydrophilic, thereby enhancing the deposition of silver nano-ink on the COP surface (Figure 8c). Thereafter, silver nano-ink was inkjet printed on the COP surface with multiple coatings to evaluate the density of silver nano-ink needed to impart antibacterial properties (Figure 8d).

#### Antibacterial Activity and Ecotoxicity Analysis of Silver Nano-Ink Printed Polymer

**Figure 9:**
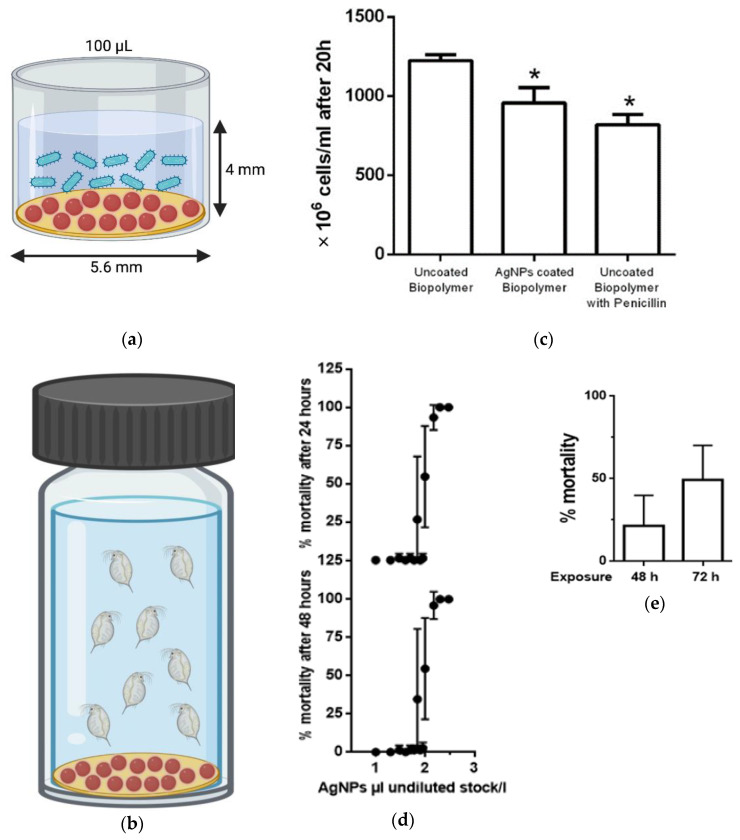
Characteristic of the experimental well used in the testing of antibacterial (**a**) and ecotoxicity (**b**) analysis including AgNPs-coated COP and organisms in suspension. (**c**) Impact of silver nano-ink printed cyclo-olefin polymer on bacterial growth. Data represent average ± SD (*n* = 4) and considered statistically significant (*) compared with uncoated polymer by Student’s *t*-test (*p* < 0.05). Mortality of daphniids upon exposure to silver nanoparticles in (**d**) suspension and (**e**) on silver nano-ink-coated cyclo-olefin polymer. Data represent average ± SD (N = 5).

Immersion antibacterial assay of silver nano-ink cyclo-olefin polymer was tested using *E. coli* cells in 96-well plates. A schematic displaying the dimensions of a single well is portrayed in Figure 9a. It was observed that the silver nano-ink printed cyclo-olefin polymer reduced the bacterial cell population from 1235 (±45) × 10^6^ cell/mL to 960 (±110) × 10^6^ cell/mL (Figure 9c). The bactericidal performance of silver-coated polymer was comparable to that of the positive control, i.e., penicillin-coated polymer, wherein a reduction in bacterial population from 1235 (±45) × 10^6^ cell/mL to 830 (±70) × 10^6^ cell/mL was observed (Figure 9c). The decrease in *E. coli* cells can be explained by the release of Ag ions from the silver nano-ink-coated cyclo-olefin polymer and its subsequent interaction with the bacterial cell membrane. For instance, the biologically synthesised silver nanoparticles biosorbed on the hydrogels were shown to inhibit the Gram-negative Pseudomonas fluorescens by the interaction of positively charged silver ions with the negatively charged sulphur and phosphorous groups on the outer surface of the cell membrane [5].

To study the ecological effects of the silver nano-ink-coated polymer on water ecosystem, the water flea Daphnia magna was used as a model organism. Daphniids have gained significant interest mainly because of their geographical distribution and pivotal role in freshwater food webs. Recently, daphniids were classified as a key indicator species in molecular ecotoxicology [24]. With nanomaterials being present in the environment and nano-silver being the most widely used nanomaterials in consumer products, the mortality rate of daphniids upon exposure to silver nano-ink was measured in a dose-dependent manner. Toxicity curves at 24 and 48 h of exposure (Figure 9d) of AgNPs in suspension showed a value of EC_50_ for 24 and 48 h of 100 μL AgNPs/L. AgNPs coated on biopolymer showed a time-dependent mortality of 23 (±17)% after 48 h and 49 (±22)% after 72 h of exposure (Figure 9e). Daphniids are well-known for their sensitivity toward the toxic effects of the materials. For instance, when tested on Daphniids, the green ionic liquids proposed as a replacement for organic solvents were found to be more toxic than conventional organic solvents [25]. Thus, the mortality observed at 48–72 h reveals the toxic effect of the AgNPs nanocoatings produced on Daphnia magna.

### 2.5. Comparison with State-of-the-Art

LaSiS and USP are the techniques often considered for nanoparticle generation under the green synthesis route. Sreenilayam et al. (2022) [9] recently showed that the LaSiS technique operated under batch mode with advanced process control could yield AgNPs size ranging between 27 and 39 nm. Though in the present study, the LaSiS operated under batch mode without any control system produced AgNPs of 45 (±40) nm, the performance of LASiS under recirculatory mode was superior with a significantly smaller size distribution of 12 (±10) nm. The present study carried out by recirculating the fluid path during laser ablation paves the way for scalable production of uniformly sized AgNPs. In the case of ultrasound pyrolysis, Kaya et al. 2020 [11] produced AgNPs size ranging between 200 and 300 nm with an average size of 258 nm. This elevated AgNPs size of 258 nm compared to that of 115 nm observed in the present study can be attributed to the increased concentration of 0.1–0.3 M and 0.001–0.1 M of silver nitrate considered in the studies, respectively. The effect of silver nitrate concentration on AgNPs diameter was also reported elsewhere in the literature, where AgNPs diameter of 20 nm was produced using USP by atomising highly dilute aqueous silver nitrate solution of 10^−7^ kmol/m^3^. Despite being small, this AgNPs size of 20 nm falls short compared to that of the AgNPs produced by the LaSiS system operated under recirculation mode.

Further, it was reported that a higher AgNPs concentration of 50 mg/L was announced for developing bacteriostatic cotton fabrics produced by the functionalisation of AgNPs [5]. However, significant inhibition of *E. coli* was noticed with 30 mg/L of AgNPs produced by LaSiS operated under recirculation mode. Raza et al. (2016) [16] reported a wet chemical route for AgNPs synthesis and developed nanoparticles in size ranging between 15 and 50 nm, which resulted in a zone of inhibition of 1.5 (±0.3) mm against *E. coli.* The lower zone of inhibition observed in spite of having exceptionally smaller nanosize could be attributed to the presence of organic molecules such as citrate encapsulating the AgNPs [16]. This reduced zone of inhibition associated with AgNPs produced via the wet chemical route produces AgNPs using LaSiS, which is more attractive and efficient for real-time application.

## 3. Materials and Methods

### 3.1. Nanoparticle Production

Two methods for producing AgNPs were used in this study: Laser synthesis in solution (LASiS) and ultrasonic spray pyrolysis (USP). The nano colloids produced were compared in terms of nanoparticle morphology and antibacterial efficacy.

#### 3.1.1. Laser Synthesis in Solution (LASiS)

**Figure 10:**
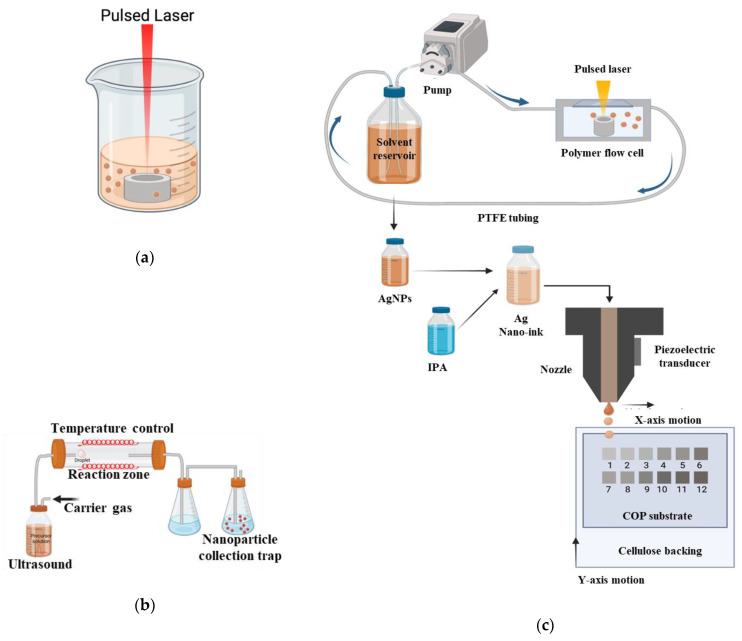
Schematic design of the (**a**) batch LASiS setup including beaker, solvent and target material, (**b**) ultrasonic spray pyrolysis technique and (**c**) recirculatory flow-based LASiS production setup including flow-cell, target, peristaltic pump, solvent reservoir, laser beam, resulting in Ag nano-ink and the inkjet printing process on COP with the numeric digits indicating the quantity of overprints (1–12).

Silver nanoparticles were produced using batch (Figure 10a) and recirculatory flow-based (Figure 10c) by laser ablation synthesis in solution (LASiS) technique as previously described in the literature [19,20]. The laser system was a picosecond-pulsed WEDGE HF 1064 (BrightSolutions, Pavia, Italy) Nd:YAG with a wavelength of 1064 nm, maximum output power of 1.2 W, pulse-width of 700 pS and a pulse repetition rate of 10 kHz [20]. The beam was rastered using an SS-12 2D-scanning galvanometer (Raylase, Weßling, Germany) in an Archimedean spiral pattern with a 5 mm outer diameter at a velocity of 5 mm·s^−1^. The position of the target in the beam waist (spot size = 100 μm in diameter) was controlled using a M-404 4PD 1D-nanoposition stage (PI, Karlsruhe, Germany). The silver ablation target (>99.999% metal basis, Scottsdale bullion silver) was machined into 8 mm discs, 4 mm thick and mechanically polished. De-gassed and deionised water was used as the solvent for all tests.

For batch LASiS, the target material was placed in a beaker and submerged in 4 mL of DI water. For recirculatory flow LASiS, an in-house designed polymer 3D-printed flow cell described, previously described in the literature contained the target material [19,20,21]. A peristaltic pump (Millipore Ltd., Burlington, VT, USA) was used to maintain a recirculating flow of DI-water over the AG target surface at 100 mL min^−1^. The flow-cell was composed of a main structure of VeroWhitePlus (RGD835) photopolymer produced by a Stratasys Connex1 Polyjet 3D printer, an EPDM O-ring and a quartz laser window [19,26].

#### 3.1.2. Ultrasonic Spray Pyrolysis (USP)

Commercial AgNO_3_ (Sigma Aldrich—99.9%, CAS Number: 7761-88-8) powder was used as the silver precursor. The precursor solutions were prepared in deionised water by dissolving defined amounts of AgNO_3_ with concentrations varying as 0.01 M, 0.005 M, 0.002 M and 0.001 M. The typical synthesis was performed with a single-step USP equipment comprised of carrier and/or reaction gas, flow regulator, ultrasonic aerosol generator (1.7 MHz; Gapusol, RBI, France), horizontal wall heated furnace with a quartz tube reactor and the washing bottles filled with ethanol, to capture the nanoparticles (Figure 10b). Details of this equipment have been previously described in the literature [8,18]. The optimal nitrogen flow of 1.5 L/min and reaction temperature of 800 °C were fixed to assure complete decomposition of silver nitrate.

### 3.2. Characterisation Techniques

#### Nanoparticle Size and Morphology

Size dispersion of the nanoparticle colloids was analysed using NanoFlex dynamic light scattering (DLS) analysis (Microtrac Inc., Hann, Germany.) using five acquisitions per 30 s [21]. The nanoparticle size and morphology were analysed using a Philips CM20 TEM operated at 200 kV. Selected area diffraction (SAD) was carried out using a 1 µm aperture. Samples for transmission electron microscopy (TEM) analysis were prepared by deposition of particles suspended in water on Plano grids [21]. UV-Vis analysis was performed using a Biochrom Libra S22 UV-Vis spectrophotometer, with a scanning range of 200–900 nm with DI water background [20]. For both DLS and UV-Vis measurements, a one-in-ten dilution of the colloid was used to mitigate measurement saturation, and no post-filtration was performed, ensuring that the DLS and UV-Vis characterisations were representative of the production process. DLS measurement was performed with AgNPs colloid in the instrument, Zetasizer Ultra, Malvern Panalytical. FTIR analysis was carried out under attenuated total reflectance (ATR) mode using PerkinElmer FT-IR spectrometer.

Fluorescence measurement was carried out with Tecan infinite 200 plate reader, with samples transferred into a 96-well plate, emission scan with an excitation wavelength of 420 nm, an integration time of 20 μs and a gain of 100. An average was taken over 25 readings for the emission wavelength measurement range of 450–800 nm [27]. FE-SEM analysis of the surfaces was carried out using a field emission scanning electron microscope (FE-SEM, Hitachi S5500). The FE-SEM characterisation was carried out by depositing a drop of colloidal AgNPs onto a polished silicon substrate and drying it in air.

### 3.3. Nano-Ink Formulation

The quality of silver nano-ink and the manufacturing methods are crucial for high-quality printing applications. The AgNPs produced by the LASiS technique operated under the recirculatory mode were used for silver nano-ink formulation. The concentration of AgNPs for inkjet printing was maintained at 0.02 g/L. The viscosity of silver nano-ink needed for inkjet printing was formulated by blending 0 to 0.8 fractions of isopropanol into deionised water containing AgNPs. The viscosity of the blend was compared with that of the commercial ink, which is an essential consideration for inkjet printing of silver nano-ink [10]. Viscosities of the silver nano-ink suspensions were measured using an Anton Paar MCR 301 Rheometer system with a maximum torque capability of 200 mNm, resolution of 0.1 nNm and a maximum angular velocity of 628 rad/s [10].

### 3.4. Surface Coating and Inkjet Printing

The viscosity and surface tension of both inks were 10–17 mPa·s and 30–38 mN·m^−1^, respectively; these aqueous inks were tailored for a Canon Pixma iP7250 (Amstelveen, The Netherlands) inkjet printer. A new refillable PGI-550BK Black ink cartridge was obtained from 118 ink.com (Felixstowe, UK). The cartridge was filled with 20 mL silver nano-ink with its viscosity adjusted to 1.8 mPa/s by adding isopropanol to make a fraction of AgNPs containing deionised water solvent-isopropanol fraction of 18% isopropanol and 82% of AgNPs containing deionised water. Cyclo-olefin polymer (COP) with a film thickness of 188 µm (ZeonorFilm^®^ ZF14–188) was selected for the substrate (Zeon Chemical LP Company, Dusseldorf, Germany). Before printing, the COP surface underwent plasma polymerisation with the plasma chamber opeating at 3 W for 5 s bursts to ensure that it had the necessary hydrophilic surface properties for printing [9]. The COP was taped onto an 80 g/m^2^ printing paper, and 12 Squares of 2 × 2 cm were printed with each square signifying the number of repeat prints on that surface as illustrated in Figure 8.

### 3.5. Antibacterial Activity and Ecotoxicity Test

#### 3.5.1. Agar Disc Diffusion Test for Antibacterial Activity of Silver Nanoparticles

The AgNPs colloids were first concentrated to a concentration of 0.3 mg/mL for the antibacterial activity tests. Standard agar diffusion tests were completed as follows: DH5-α *E. coli* cells (Invitrogen, San Diego, CA, USA) were cultured in Luria Bertani broth for 12 h and centrifuged at 4000 rpm. The separated cells were then re-suspended in phosphate-buffered saline (PBS), with the cell concentration adjusted to 10^7^ mL^−1^. Optical density measurements were performed at 600 nm using a spectrophotometer (Biochrom Libra S22, Cambridge, UK) [16]. The bacterial cells were diluted to desired cell concentration using PBS and related optical density with cell counts using a haemocytometer under a light microscope (OPTIKA microscopes B-290 series, Ponteranica, Italy). The LB plates were prepared with 100 µL of culture. Cellulose test discs (Oxoid CT0998B) were mounted on the agar. Various concentrations of AgNPs with one-in-two (150 mg/L) and one-in-ten (30 mg/L) dilutions of concentrated AgNPs were prepared from the original 300 mg/L concentration. Accordingly, each concentration of AgNPs was deposited onto the 6 mm cellulose test discs at 20 µL volume for its subsequent antibacterial analysis. The cellulose disc deposited with 200 mg/L of commercial AgNPs procured from Sigma-Aldrich^®^ served as the positive control, and the cellulose disc with deionised water was used as the negative control in the present study. The plates were incubated at 37 °C for 18 h, with the inhibition ring diameter measured via a digital Vernier calliper.

#### 3.5.2. Immersion Antibacterial Activity Testing of Silver Nanoink-Printed Polymer

Bacteria (*E. coli*) were cultured in Luria-Bertani (Merck^®^, Darmstadt, Germany) broth at 30 °C under agitation (200 rpm). Bacteria were pelleted (3000× *g*, 4 °C, 5 min), washed in sterile phosphate buffer saline (PBS) and pelleted twice. A dilution of bacteria in PBS was prepared at a concentration of 2300 cells/mL, and 100 μL was exposed to AgNPs coated on a biopolymer film (approximate dimension of 0.25 cm^2^) and incubated for 2 h at 30 °C under agitation (200 rpm). After exposure to PBS, 900 μL LB media were added, and surviving bacteria were allowed to grow for 20 h under agitation (200 rpm). Growth was measured by absorbance at 600 nm and converted to cell concentration [4]. For comparisons of growth inhibition, penicillin G (50 μM) was incubated in the presence of the uncoated biopolymer.

#### 3.5.3. Ecotoxicological Effects of Silver Printed Polymer

Daphniids were cultured in conformity with OECD guidelines in 4-litres beakers in OECD media (final concentrations 0.29 g CaCl_2_.2H_2_O /L, 0.123 g MgSO_4_.7H_2_O/L, 0.065 g NaHCO_3_/L, 0.0058 g KCl/L, 2 μg Na_2_SeO_3_/L, pH 7.7) using 16 h:8 h of light and dark photoperiod at 20 °C and a density of 100 adults per 4 litres of media [28]. Neonates (<24 h) were collected and exposed to 50 mL OECD media against unexposed controls, and mortality was measured after 24 and 48 h of exposure.

### 3.6. Statistical Analysis

The samples were analysed in triplicate, and the same was used for statistical analysis. Data were analysed and plotted using Excel software, and Student’s t-test analysis was performed using the GraphPad^®^ Prism software. Results were expressed as average ± standard deviation (±SD) and considered statistically significant for *p* < 0.05.

## 4. Conclusions

Silver nanoparticles were produced sustainably by employing ultrasound pyrolysis and pulsed laser ablation technique operated under batch and recirculation mode. However, a difference in particle size was observed, particularly LaSiS-operated under recirculation mode yielded smaller AgNPs of 12 (±10) nm with narrow size distribution. The smaller size of silver nanoparticles aided in attaining better antibacterial activity against *E.coli* cells. Silver nano-ink containing 18% isopropanol and 82% silver nanoparticles containing deionised water were optimal and displayed a viscosity equivalent to that of commercial ink. Though the strong reduction of *E. coli* cells portrays the antibacterial property, the mortality of daphniids cells demonstrates that the silver nano-ink-coated cyclo-olefin polymer is toxic. Thus, the present study illustrates the routes for synthesising AgNPs with antibacterial properties. Further, the inkjet printing of silver nano ink on the outer surface of the polymeric surface minimises the migration of AgNPs into the food system. However, as results showed, exposure of the AgNPs coatings to *Daphnia magna* proved detrimental with a 49% mortality rate after 72 h. This indicates the need for caution when producing nanofilms that may end life in a water system. In addition, additional studies on AgNPs release and the role of AgNPs in enhancing the barrier properties of packaging material and the shelf life of food products are warranted.

## Figures and Tables

**Table 1:** Comparison of the size of AgNPs produced through different methods and the zone of inhibition exhibited by AgNPs on *E. coli* cells.

Production Method	AgNPs Diameter	Tested AgNPs Concentration	Zone of Inhibition (ZOI) Diameter
Ultrasound pyrolysis (USP)	115 nm	300 mg/L	6.34 mm
		150 mg/L	6.15 mm
		3 mg/L	5.59 mm
Batch LaSiS	45 nm	300 mg/L	6.31 mm
		150 mg/L	6.24 mm
		3 mg/L	5.91 mm
Recirculatory LASiS	12 nm	300 mg/L	6.45 mm
		150 mg/L	5.86 mm
		3 mg/L	5.72 mm
Commercial AgNPs (Positive control)	10 nm	20 mg/L	7.15 mm
Deionised water (Negative control)	Not Applicable		0 mm

## Data Availability

Not applicable.
